# Lea’s Series of Medical Epitomes

**Published:** 1904-02

**Authors:** 


Lea's Series of Medical Epitomes. Wathen's Epitome of Histology. A Manual for Students and Physicians. By John R. Wathen, A. M., M. D., Professor of Surgery, etc., formerly Professor of Histology and Pathology, Kentucky School of Medicine, Louisville, Ky. 12mo, 220 pages, 114 illustrations. Cloth, $1.00 net. Lea Brothers & Co., Publishers, Philadelphia and New York. 1903.

This book contains a wealth of valuable information on the subject, and that, too, in a very practical form. The chapter on the technique of preparing and staining tissue is well worth the price of the book.

